# A Comparative Transcriptome Analysis of *Volvariella volvacea* Identified the Candidate Genes Involved in Fast Growth at the Mycelial Growth Stage

**DOI:** 10.3390/genes11020161

**Published:** 2020-02-04

**Authors:** Ming Liu, Ting Yu, Puneet Kumar Singh, Qinjian Liu, Hao Liu, Qingfeng Zhu, Zitian Xiao, Jiang Xu, Yangyang Peng, Shiyu Fu, Shicheng Chen, Huanqing He

**Affiliations:** 1 Vegetables Research Institute, Guangdong Academy of Agricultural Sciences, Guangdong Key Laboratory for New Technology Research of Vegetables, Guangzhou 510640, China; liuming@gdaas.cn (M.L.); xiaozitian@gdaas.cn (Z.X.); xujiang@gdaas.cn (J.X.); pengyangyang@gdaas.cn (Y.P.); 2 Agro-Biological Gene Research Center, Guangdong Academy of Agricultural Sciences, Guangzhou 510640, China; yuting@gdaas.cn (T.Y.); liuqinjian@gdaas.cn (Q.L.); zhuqingfeng@gdaas.cn (Q.Z.); 3 School of Light Industry and Engineering, South China University of Technology, Guangzhou 510640, China; Puneet@scut.edu.cn (P.K.S.); feliuh@scut.edu.cn (H.L.); shiyfu@scut.edu.cn (S.F.); 4 Department of Microbiology and Molecular Genetics, Michigan State University, East Lansing, MI 48824, USA

**Keywords:** *Volvariella volvacea*, transcriptome, growth performance

## Abstract

The edible straw mushroom, *Volvariella volvacea*, is one of the most important cultivated mushrooms in tropical and sub-tropical regions. Strain improvement for *V. volvacea* is difficult because of the unknown mechanisms involved in its growth regulation and substrate utilization. A comparative physiological and transcriptomic study was conducted between two commercially available straw mushroom strains (v9 and v26) to explore their fast-growth regulation mechanism(s). The physiological study showed that *V. volvacea* v9 had a shorter growth cycle and higher biological efficiency (4% higher) than that in v26. At least 14,556 unigenes were obtained from the four cDNA libraries (two replicates per strain). Among them, the expression of 1597 unigenes was up-regulated while 1352 were down-regulated. Four heat-shock proteins were highly expressed in v9, showing that v9 has the better ability to handle stresses and/or environmental changes. Moreover, up to 14 putative transporter genes were expressed at a higher level in v9 than those in v26, implying that v9 has a better ability to transport nutrients or export xenobiotics efficiently. Our report allows to identify the candidate genes involved in the fast growth requirement of *V. volvacea*, which represents a valuable resource for strain improvement in this commercially important edible mushroom.

## 1. Introduction

Straw mushroom (*Volvariella volvacea*), also called Chinese mushroom, has a long history of cultivation in tropical and subtropical regions [1,2]. In China, at least 330,000 tons of straw mushroom were produced in 2010 (accounting for more than 80% of the global production). The yield ranked the fifth among the commercially cultivated mushrooms [3]. Besides its high nutrient value, *V. volvacea* also has medicinal importance, including antitumor polysaccharides, immunosuppressive proteins and immunomodulatory lectins [4,5]. Despite its high demand in the mushroom markets, the conversion efficiency of culture substrates into fungal fruit bodies is remarkably lower than those major cultivated species [6]. 

During the past decade, with the rapid development of high-throughput sequencing technologies, genome-wide expression analysis has been used for investigating genes involved in lignocellulose decomposition, secondary metabolites and fruiting body development in various edible mushrooms, including *Schizophyllum commune* [7], *Ganoderma lucidum* [8], *Agaricus bisporus* [9] and *Lentinula edodes* [10]. In *V. volvacea* [3,11,12], the genes responsible for tolerance of low temperature [13,14], the mating-type system and fruit-body development were also explored by comparative transcriptome analysis techniques [15]. 

Edible fungi with fast growth features are associated with a short growth cycle, a better substrate utilization efficiency and higher yields. However, the molecular mechanisms involved in *V. volvacea* fast-growth remain unexplored. Previous studies showed that the fast-growing strains showed higher activities of endoglucanase, laccase and polyphenol oxidase when cultivated on pasteurized substrates; further, a higher xylanase and β-glucosidase were found in mushroom when the composted substrates were used [16,17]. Fast-growing fungal strains utilize the mitogen-activated protein kinase (MAPK) pathways for mating, morphogenesis, stress tolerance and fruiting-body development. In this study, we conducted a comparative transcriptome analysis between a faster growing *V. volvacea* strain, v9, and a slower growing strain, v26, to identify the candidate genes involved in fast growth at the mycelial growth stage. 

## 2. Results

### 2.1. Comparations of the Growth Rates between V. volvacea Strain v9 and Strain v26 

**Figure 1:**
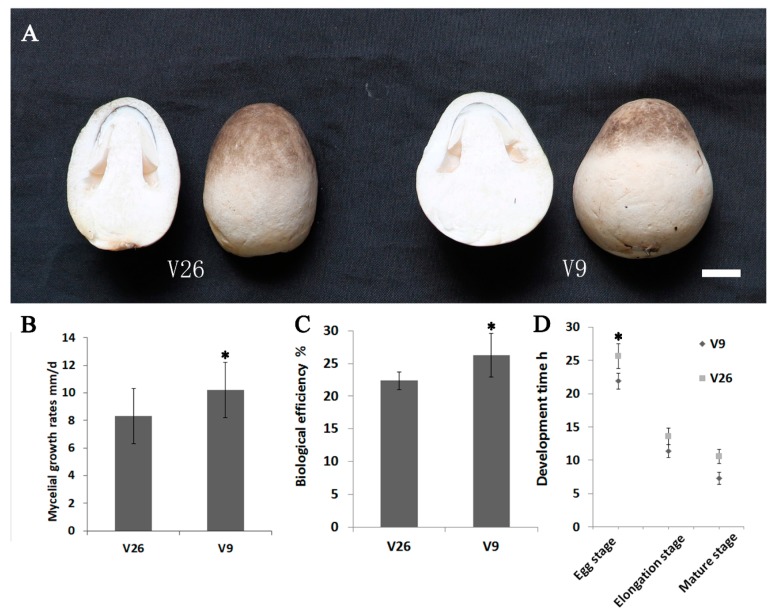
Differentiating characteristics of *V. volvacea* strains v26 and v9. (**A**) The “egg” stage in the development of *V. volvacea*. Note that the v26 fruiting body is oval-shaped, whereas v9 is bell-shaped. (**B**) Mycelial growth rates of *V. volvacea* strains v26 and v9. (**C**) The biological efficiency of v26 and v9. Both strains were cultivated on rice straw and cotton waste. (**D**) Developmental periods of v26 and v9. Strain v9 developed faster than v26. Each experiment was repeated three times. Mycelial growth rates, biological efficiency and developmental stages time were carried out by a Student’s *t* test. The mycelial growth rates, biological efficiency and egg stage time were significantly different (*p* < 0.05, The elongation stage and mature stage developmental time were not significantly different (*p* > 0.05). Bar = 1 cm in (**A**). Data are presented as the average ± SD in (**B**–**D**).

*V. volvacea* v9 had a bell-shaped fruit body with a gray-white color. It was noted that its basal surface contacted closely to the cultivated materials. However, the fruit body of strain v26 was oval-shaped with a gray color. The contact area between the fruiting body and the cultivated materials was smaller than that of v9 (Figure 1A). On the PDA media, mycelial growth rates of v9 and v26 were 10.2 ± 2 mm/d and 8.3 ± 2 mm/d, respectively (Figure 1B). The following formula for biological efficiency was used: biological efficiency (%) = fresh fruit body weight/substrate dry weight × 100. The biological efficiency of strain v9 was 26.3%, which was higher than strain v26 (22.3%) (Figure 1C). Three development stage periods were measured (egg stage, elongation stage and mature stage) [18]. In the egg stage, the stipe was hidden and ovoid; in the elongation stage, the stipe was stretched out of the universal veil and the pileus was not opened; in the mature stage, the pileus was fully expanded. The egg stage, elongation stage and mature stage time in strain v9 was 21.9, 11.5 and 7.3 h, respectively. Instead, the mature time for strain v26 was 25.6, 13.6 and 10.5 h, respectively (Figure 1D). Collectively, v9 had higher biological efficiency and a shorter mature time than v26.

### 2.2. Sequencing, Assembly and Functional Annotation of V. volvacea

The four cDNA libraries from the mycelial growth stage yielded a total of 67 million raw reads. Up to 16 million reads per sample were obtained after the data filtering and trimming (Table 1). A total of 14,556 unigenes were assembled for all samples (Table 1). Among them, at least 85% of the unigenes were annotated using the databases of NR and KEGG (Table 1). A total of 58% and 43% of the unigenes encoded the “hypothetical proteins” in strains v9 and v26, respectively. The above observation agreed that many genes in *V. volvacea* were not able to be assigned to the known functions in Basidiomycetes [7,9].

**Figure 2:**
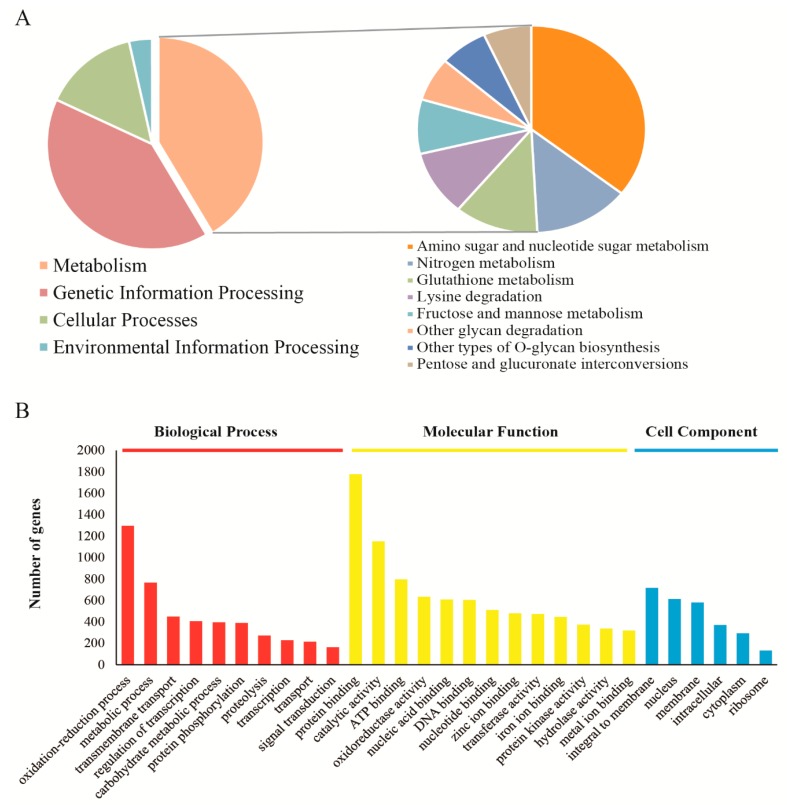
Functional annotation of *V. volvacea*. (**A**) Pie chart showing the percent of genes enriched on the KEGG pathways from transcriptome data. (**B**) GO annotation of unigenes, including the biological process, molecular function and cell component.

Most of the unigenes were enriched in “Metabolism” and “Genetic Information Processing” (Figure 2A, left panel) using the KEGG pathway enrichment analysis. Among the metabolism pathways, the amino sugar and nucleotide sugar metabolism were most abundant, followed by nitrogen metabolism, glutathione metabolism, lysine degradation and fructose and mannose metabolism (Figure 2A, right panel). Enrichment analysis of Gene Ontology (GO) showed that “Oxidation-reduction process” had the most abundant genes. Moreover, a large number of genes involved in the “Carbohydrate Metabolic Process” and “Proteolysis” were found in the “Biological Process” (Figure 2B), indicating that *V. volvacea* has a strong potential to utilize plant-derived culture substrates.

### 2.3. Gene Expression and Identification of DEGs

**Figure 3:**
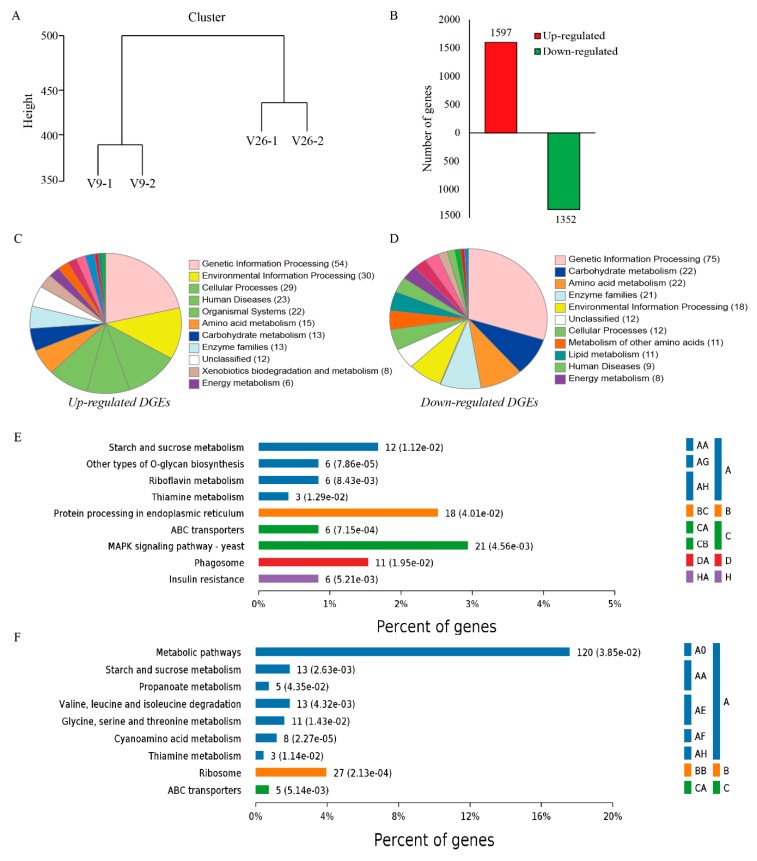
The analysis of gene expression. (**A**) The expression patterns from all samples were clustered. (**B**) The number of up/down-regulated DEGs were showed when v9 compared with v26. (**C**) The functional categories of the KEGG pathway enriched by up-regulated DEGs are showed. (**D**) The functional categories of the KEGG pathway enriched by down-regulated DEGs are displayed. (**E**) The pathways were significantly enriched by up-regulated DEGs (*P* (adj.) < 0.05). (**F**) The pathways were significantly enriched by down-regulated DEGs (*P* (adj.) < 0.05). **A**: Metabolism, **B**: Genetic Information Processing, **C**: Environmental Information Processing, **D**: Cellular Processes, **H**: Other and unknown. A0: Global and overview maps; AA: Carbohydrate metabolism; AE: Amino acid metabolism; AF: Metabolism of other amino acids; AG: Glycan biosynthesis and metabolism; AH: Metabolism of cofactors and vitamins; BB: Translation; BC: Folding, sorting and degradation; CA: Membrane transport; CB: Signal transduction; DA: Transport and catabolism; HA: Other and unknown.

At least 13,488 (92.7%), 13,427 (92.2%), 13,200 (90.7%) and 12,955 (89%) transcripts were identified in v9-1, v9-2, v26-1 and v26-2 libraries, respectively. The cluster analysis showed that v9-1 was close to v9-2 and v26-1 was clustered with v26-2. Instead, v9-1 and v9-2 separated with v26-1 and v26-2. This observation indicated that the gene expression patterns were different in the two *V. volvacea* strains (Figure 3A). qRT-PCR analysis of the selected genes was performed to validate the transcriptome data. The same gene expression patterns were consistent with those by the transcriptome analysis (Supplementary Table S1). 

At least 2949 transcripts were shared between v9 and v26 at the vegetative growth stage. Among them, the expression of 1597 unigenes was up-regulated while 1352 were down-regulated (Figure 3B). Up-regulated DEGs were assigned to Genetic Information Processing by the KEGG enrichment analysis of DGEs, followed by Environmental Information Processing, Cellular Processes, Amino Acid Metabolism and Carbohydrate Metabolism, as well as several others (Figure 3C). Down-regulated DGEs were enriched in Genetic Information Processing, followed by Carbohydrate Metabolism, Amino Acid Metabolism, Enzyme Families, Environmental Information Processing and Cellular Processes (Figure 3D). 

A further significant enrichment analysis of the KEGG pathway showed that both up-regulated DGEs and down-regulated DGEs were significantly enriched on the pathways of Starch and Sucrose Metabolism, Thiamine Metabolism and ABC Transporters (Figure 3E,F). Moreover, up-regulated DGEs were significantly enriched in the MAPK signaling pathway within Environmental Information Processing and Phagosome within cellular processes (Figure 3E). However, down-regulated DGEs were significantly enriched in the Propanoate Metabolism within Carbohydrate Metabolism, and pathways related to Amino Acid Metabolism of Valine, Leucine and Isoleucine Degradation, Glycine, Serine and Threonine Metabolism as well as Cyanoamino Acid Metabolism (Figure 3F). 

Thiamine plays a vital role in the metabolism of glucose and energy production. At least six genes involved in “Thiamine Metabolism” pathways were investigated in detail because they were significantly enriched in the KEGG pathways (Figure 3E,F). The expression level of thiamine synthase genes *iscS* (ctg4698_g1) was higher in v9 than in that in v26, demonstrating a more active vitamin B1 synthesis activity. The thiamine metabolism gene *THI20* (ctg9033_g1) and *THI4* (ctg9315_g1) in v9 showed a lower expression level than that in v26. The expression of other selected thiamine metabolism genes was only slightly different between the strains. 

The carbohydrate utilization gene is expressed differently in the two strains (Table 2). Three genes encoding the cellulase 1,4-beta-cellobiosidase (CBH1 and CBH2) were upregulated in v9, and two of the three lytic polysaccharide monooxygenase genes (*LPMO*s) were expressed at a higher level in v9. Moreover, at least 8 β-glycosidase genes (bglX) were downregulated in v9 compared to v26. Two (ctg9698_g1 and ctg11581_g1) of the three selected α-amylase genes (*AMY*) showed a higher expression level in v9 than they did in v26 (Table 2).

### 2.4. Genes Involved in MAPK Signaling Pathway

At least 21 up-regulated and 10 down-regulated unigenes were discovered in the MAPK signaling pathway. These 31 unigenes were distributed in four pipelines of the MAPK signaling pathway (yeast), including pheromone to mating, cell wall stress to remodeling, high osmolarity to osmolyte synthesis and starvation to filamentation (Table 3). Genes encoding BEM1 (K11237, bud emergence protein 1), SWE1 (K03114, mitosis inhibitor protein kinase SWE1), TUP1 (K06666, general transcriptional corepressor) and negative-regulated CDC42 gene RGA1_2 (K19839, Rho-type GTPase-activating protein 1/2) were highly expressed in v9 compared to v26. This indicated that the growth-related genes were more actively transcribed in v9 than in v26. Instead, genes involved in proliferation, such as CDC42 (K04393, cell division control protein 42), STE3 (K04627, pheromone a factor receptor), BCK1 (K11229, mitogen-activated protein kinase), CTT1 (K03781, catalase) and TEAD (K09448, transcriptional enhancer factor) were expressed more in v26 than in v9. 

### 2.5. String Analysis 

**Figure 4:**
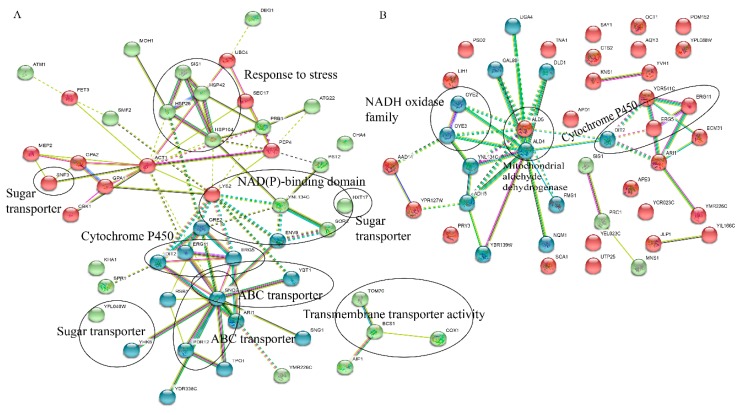
STRING analysis. A protein interactive network was used to display the genes that were related to the highly up-regulated, differently expressed unigenes (**A**), or related to the highly down-regulated, differently expressed unigenes (**B**). Gene products are represented with circles and known associations between each gene or gene product are represented with a connecting line. Nodes of genes participating in similar functions are circled in red or in blue or in green. The network cluster was based on the k-means clustering method.

A total of 262 highly up-regulated DEGs and 302 highly down-regulated DEGs were obtained using string analysis (FDR < 0.001). A total of 58 up-regulated unigenes was annotated to 48 and 55 down-regulated unigenes was annotated to 44 (Figure 4). As shown in Figure 4A, up-regulated genes could be grouped into heat-shock proteins (HSPs), sugar transporters, ABC transporters, cytochrome P450, NAD(P)-binding domain and others (Figure 4A). Four heat-shock proteins, including HSP26, HSP104, HSP42 and SIS1 (Type II HSP40 co-chaperone), were highly expressed in v9 (FPKM > 1400) while those in v26 were not (FPKM < 700). Fourteen putative transporters were expressed at a higher level in v9 than those in v26. These transporters seem to be involved in various nutrient transportation; for example, four sugar transporters were found: HXT17 (hexose transporter), SNF3 (low glucose sensor that regulates glucose transport), YFL040W (transporter member of the sugar porter family) and YHK8 (presumed antiporter of the major facilitator superfamily); four ABC transporters were found: ATM1 (exports mitochondrially synthesized precursors of iron-sulfur (Fe/S) clusters to the cytosol), PDR12 (weak-acid-inducible multidrug transporter), SNQ2 (multidrug transporter) and YBT1 (bile acid transport); as well as other transporters were found: BCS1 (AAA ATPase family), COX1 (subunit I of cytochrome c oxidase), RSB1 (transport sphingoid long chain base (LCB)), SMF2 (manganese transporter), TOM70 (transit peptide receptor) and TPO1 (polyamine transporter that recognizes spermine, putrescine, and spermidine). Moreover, six up-regulated and five down-regulated unigenes were significantly enriched in the pathway of the ABC transporters (Table 4). The expression of gene ABCB1 (K05658, ATP-binding cassette, subfamily B (MDR/TAP), member 1) was higher in v9 than in v26. ABCB1 encoded the ATP-binding ATPases, which played important roles in the exportation of xenobiotics. Therefore, one can assume that v9 may more actively export xenobiotic molecules than v26.

## 3. Discussion

The current cultivation mode of straw mushrooms are field cultivation and indoor cultivation [2,19,20]. Strain v9 is more appropriate for cultivation in the field while v26 is mainly cultured indoor for commercial production. Compared to the indoor cultivation, the filed cultivation mode encounters more dramatic temperature changes between day and night. Temperature fluctuations lead to a series of metabolism activity adaptions; for example, changes in unsaturated fatty acid biosynthesis, as well as the accumulation of trehalose and glycogen. The cold-shock and heat-shock proteins played important physiological roles during the temperature fluctuations periods [21,22]. It is well known that the production of heat shock proteins are promoted by abnormal stress factors in various fungi. Heat-shock proteins function as molecular chaperone that participate in refolding or degradation of stress-damaged proteins [23]. For example, exposure to low temperature (i.e., 4 °C) for more than eight hours will cause irreversible damage to the *V. volvacea* mycelium [24]. To respond to the dramatic temperature, a number of *HSP* genes in *V. volvacea* were down-regulated after low temperature exposure [1]. In this study, we also found that the expression levels of *hsp104*, *hsp42* and *hsp26* genes in v9 was much higher than those in v26 (Figure 4). High expression of heat-shock proteins in v9 may enhances its adaptability to temperature fluctuations. This may be one of the reasons why the v9 strain was selected for field cultivation

The mycelium growth rate of the v9 strain is faster than that of the v26 strain, and the biological efficiency is higher (Figure 1). The mycelial growth performance and biological efficiency of a mushroom species is mainly attributed to its hydrolytic enzymes system [25,26]. *V. volvacea* had a higher number of enzymes related to the degradation of cellulose, hemicellulose and pectin compared with other basidiomycetes [3,20,27]. Our results showed several carbohydrate utilization genes in v9 was transcribed at higher levels than those in v26; for example, cellulase 1,4-β-cellobiosidase (CBH1 and CBH2), which may efficiently contribute to cellobiose releasing from cellulose. Cellobiose was an inducer for the expression of cellulase genes (e.g., 1,4-β-cellobiosidase genes) in many fungi, including *T. reesei* and *V. volvacea* [28,29]. LPMOs are involved in oxidatively cleaving glycosidic linkages, which leads the substrate to be more susceptible to hydrolysis by those conventional cellulases [30]. Further, β-glycosidase genes (*bglX*) were downregulated in v9. β-glycosidases release glucose from cellobiose, which represses the transcription of cellulase genes [31,32]. Such a regulation mechanism may enable v9 to maintain a higher level of expression of extracellular cellulase. Similarly, the β-D-xylosidase gene *XYL4* was expressed at a higher level in v26 than it did in v9. β-D-xylosidases digested xylobiose to produce glucose, which is a repressor for xylanase gene expression [31,32]. Two (ctg9698_g1 and ctg11581_g1) of the three selected α-amylase genes (*AMY*) showed a higher expression level in v9 than they did in v26 (Table 2), indicating that these two *AMY* gene was possibly involved in starch utilization in the fast-growth stage. The hexokinase gene *HK* (ctg4916_g1) transcription level in v9 was around 4-fold higher than in v26, indicating that v9 was able to metabolize and transform the simple soluble sugars (e.g., glucose or fructose) into energy for the fast growth. Moreover, two *TREH* genes for the conversion of trehalose to glucose were differently regulated (ctg9797_g3 was upregulated in v9). The transcriptional level of *malZ* showed a slightly higher expression in v26 than it did in v9, which indicated a faster conversion of maltose into glucose in v26 than in v9. The higher expression of these carbohydrate utilization genes may contribute to a faster mycelium growth rate and higher biological efficiency of v9.

Our quantitative analysis and comparison of gene expression profiles in two strains suggested that MAPK modules might be involved in mycelial growth in *V. volvacea*. However, it remains unclear what the exact role of these MAPK pathway genes are; identifying potential regulators and targets in MAPK pathways will provide insights into the signal transduction pathways of *V. volvacea*. Our data also revealed that gene expression profiles were different between v9 and v 26 when MAPK signaling pathway analysis was performed (Table 3). MAPK modules serve central roles in intracellular signal transduction and are evolutionary conserved in eukaryotic cells, including yeast and fungi [33,34]. The MAPK cascade are involved in mating and pathogenic development in *Ustilago maydis* [35]. In *Lentinula edodes*, the MAPK cascade was also reported to play important roles in light signal transmission and metabolic regulation [36]. 

We have found an array of genes that contribute to the growth of *V. volvacea* and should increase our understanding of the fundamental and cellular processes of *V. volvacea* for commercial production and industrial use. 

## 4. Materials and Methods 

### 4.1. Strains and Culture Conditions 

The *V. volvacea* dikaryotic strain v26 was isolated in a greenhouse from Foshan Zhihua Fungus Co., Ltd. (Foshan, China), and v9 was isolated in a field from Zhongshan Minzhong Edible Fungus Cooperative; both were preserved in the Fungal Culture Collection of Guangdong Key Laboratory for New Technology Research of Vegetables. The microorganisms used in the current study were maintained on potato dextrose agar (PDA, Merck, Germany) at 30 °C. The mycelial growth rate was determined in a Petri dish with a diameter of 9 cm. A 0.5 cm^2^ mycelial agar plug was transferred into the center of a fresh PDA plate and the colonies’ radii were measured every 24 h. Straw mushroom strains were cultivated on mixed substrates including 50% rice straw and 50% waste cotton (each with 65% water content) at 30 °C. Various developmental stages of *V. volvacea* were differentiated using the methods described previously by Chang and Yau [18]. At least 10 replicates for each stage were analyzed. 

### 4.2. RNA Extraction and cDNA Library Construction and Sequencing

*V. volvacea* strains v9 and v26 were cultured in 250 mL flasks containing 100 mL Potato Dextrose Broth (PDB) at 30 °C for 5 days. Mycelium were collected and immediately stored in liquid nitrogen. The two replicates per strain were used to construct the comparative cDNA libraries. Total RNA was extracted using Trizol reagents. A total of 1 μg of RNA was proceeded with the Illumina TruSeq RNA Sample Preparation kit v2, including steps to purify and fragment the mRNA, carry out first-strand cDNA synthesis and second-strand cDNA synthesis, repair the ends, adenylate the 3’ ends and ligate the adaptors. Following PCR amplification, the libraries were validated using an Agilent 2100 bioanalyzer (Agilent, Santa Clara, CA 95051, USA), which indicated average cDNA fragments of ~360 bp in length.

Four cDNA libraries were sequenced using an Illumina MiSeq sequencing platform (available at Agro-biological Gene Research Center of Guangdong Academy of Agricultural Sciences, Guangzhou, China) by using a 2× 75 bp MiSeq reagent kit v3 (Illumina, San Diego, CA 92122, USA). Raw paired-end reads were submitted to the Sequence Read Archive of the NCBI (accession number: PRJNA408191).

To examine the expression of the 14 putative thiamine metabolism- as well as starch and sucrose metabolism-related genes, qRT-PCR assays were performed. The primers for the 14 genes related to thiamine metabolism, starch and sucrose metabolism are listed in Supplementary Table S1. The PCR mix was prepared with 1 μL cDNA samples as templates in the presence of a SYBR Green PCR Master Mix and gene-specific primers. The reactions were performed in a BIO-RAD Cycler IQ Multi-Color Real Time PCR Detection System (Bio-Rad, Hercules, CA, USA). The relative levels of the amplified mRNA were evaluated according to the 2^−ΔΔCt^ method using the actin gene for normalization.

### 4.3. Sequence Processing and Bioinformatics Analysis

To obtain high quality sequences, the QC Toolkit (v2.3.1) was adopted to remove adaptor sequences, ambiguous reads and low-quality reads [37]. The clean reads from all samples were pooled together to perform the de novo assembly using the Trinity program (r20140717), which included three modules, namely inchworm, chrysalis and butterfly [38], with parameters of “--seqType fq --JM 50G --CPU 12 --trimmomatic”. Then the nucleotide sequences of the unigenes were aligned to the databases of NR and KEGG using the Blastx program with the e-value lower than 1e-5. Moreover, the clean reads per sample were aligned to the assembled transcripts with using Bowtie [39]. RSEM [40] was used to calculate the FPKM values (Fragments Per kb per Million reads) to obtain gene expression level, which were done through a perl script (align_and_estimate_abundance.pl --transcripts Trinity.fasta --left SeqR1.fq --right SeqR2.fq --seqType fq --est_method RSEM --aln_method bowtie --trinity_mode --prep_reference). EdgeR [41] was performed to detect differential expression with an FDR ≤ 0.05 and a relative change threshold of 2-fold. KOBAS [42] was used for enrichment analysis of the Gene Ontology and KEGG pathway. The functional differences between the two strains were considered significant using Benjamini–Hochberg FDR (corrected *p* < 0.05). String analysis [43] was carried out to show the protein interactive network. Figures were plotted in the R environment (v3.1.2) (http://www.r-project.org).

## Figures and Tables

**Table 1:** The data characteristics.

Sequencing	Raw Reads	67,399,626
	Clean reads	65,013,746
Assembly	Unigenes	14,556
	N50	2461
	Max length	16,297
	Min length	201
	Average length	1410
	Total assembled bases	20,524,297
Annotation	NR	10,890 (86%)
	KEGG	10,714 (85%)

**Table 2:** DGEs enriched in the starch and sucrose metabolism pathways.

Unigene ID	Expression							KEGG			qPCR
	v9-1	v9-2	v26-1	v26-2	Log2FoldChange	FDR	Status	KO	Description	Gene Name	
ctg4916_g1	98.51	90.22	20.4	27.54	2.02721	3.76 × 10^−31^	up-regulated	K00844	hexokinase [EC:2.7.1.1]	HK	4.51/1
ctg7672_g1	20.81	19.75	8.6	8.55	1.28427	1.04 × 10^−36^	up-regulated	K01178	glucoamylase [EC:3.2.1.3]	SGA1	
ctg164_g1	0.99	0.78	23.19	23	−4.7117	1.13 × 10^−147^	down-regulated	K01178	glucoamylase [EC:3.2.1.3]	SGA1	
ctg2341_g1	3.76	2.34	1.03	1.36	1.34333	9.35 × 10^−2^	up-regulated	K01178	glucoamylase [EC:3.2.1.3]	SGA1	2.23/1
ctg9698_g1	39.85	34.04	15.43	16.83	1.17589	3.42 × 10^−20^	up-regulated	K01176	alpha-amylase [EC:3.2.1.1]	AMY	
ctg11581_g1	271.23	249.79	117.41	125.9	1.0762	7.15 × 10^−95^	up-regulated	K01176	alpha-amylase [EC:3.2.1.1]	AMY	1.99/1
ctg8677_g2	3.57	3.94	11.08	10.41	−1.5319	7.84 × 10^−18^	down-regulated	K01176	alpha-amylase [EC:3.2.1.1]	AMY	
ctg3960_g1	6.87	6.38	2.19	1.84	1.69059	1.47 × 10^−14^	up-regulated	K01225	cellulose 1,4-beta-cellobiosidase [EC:3.2.1.91]	CBH1	2.29/1
ctg10469_g1	15.91	16.43	7.61	5.71	1.34346	9.80 × 10^−19^	up-regulated	K19668	cellulose 1,4-beta-cellobiosidase [EC:3.2.1.91]	CBH2	
ctg7134_g2	5.73	6.07	0.43	4.8	2.21162	1.50 × 10^−5^	up-regulated	K19668	cellulose 1,4-beta-cellobiosidase [EC:3.2.1.91]	CBH2	1.30/1
ctg7443_g2	4.9	3.55	1.59	0.74	1.79673	8.55 × 10^−2^	up-regulated	K19356	lytic cellulose monooxygenase (C1-hydroxylating) [EC:1.14.99.54]	E1.14.99.54	1.44/1
ctg2646_g1	3.45	2.9	0.18	0	4.69409	6.75 × 10^−8^	up-regulated	K19356	lytic cellulose monooxygenase (C1-hydroxylating) [EC:1.14.99.54]	E1.14.99.54	2.43/1
ctg2256_g1	0	0	2.88	2	−5.995	1.02 × 10^−23^	down-regulated	K19356	lytic cellulose monooxygenase (C1-hydroxylating) [EC:1.14.99.54]	E1.14.99.54	
ctg10311_g1	2.74	3.01	17.87	17.93	−2.6469	6.07 × 10^−55^	down-regulated	K01187	alpha-glucosidase [EC:3.2.1.20]	malZ	
ctg7514_g1	3.49	1.64	1.08	1.04	1.31816	4.9 × 10^−2^	up-regulated	K05349	beta-glucosidase [EC:3.2.1.21]	bglX	1.86/1
ctg5449_g1	1.38	1.25	3.45	2.69	−1.241	1.35 × 10^−3^	down-regulated	K05349	beta-glucosidase [EC:3.2.1.21]	bglX	
ctg1206_g1	1.69	0	3.87	8.25	−2.6145	1.35 × 10^−2^	down-regulated	K05349	beta-glucosidase [EC:3.2.1.21]	bglX	
ctg1654_g1	0.85	0	2.33	2.26	−2.2696	4.85 × 10^−2^	down-regulated	K05349	beta-glucosidase [EC:3.2.1.21]	bglX	
ctg2108_g1	0	1.01	2.4	3.48	−2.4362	4.4 × 10^−2^	down-regulated	K05349	beta-glucosidase [EC:3.2.1.21]	bglX	
ctg9569_g1	5.18	5.16	11.9	12.34	−1.2461	5.84 × 10^−26^	down-regulated	K05349	beta-glucosidase [EC:3.2.1.21]	bglX	
ctg6015_g3	1.97	1.19	5.98	6.25	−1.9476	2.98 × 10^−11^	down-regulated	K01188	beta-glucosidase [EC:3.2.1.21]	E3.2.1.21	
ctg7541_g1	5.74	5.46	15.47	18.25	−1.5991	3.44 × 10^−32^	down-regulated	K01188	beta-glucosidase [EC:3.2.1.21]	E3.2.1.21	
ctg9797_g3	391.26	395.33	0.1	0	12.679	0	up-regulated	K01194	alpha,alpha-trehalase [EC:3.2.1.28]	TREH	1.59/1
ctg1603_g1	0	0.62	5.59	7.89	−4.1037	2.06 × 10^−5^	down-regulated	K15920	beta-D-xylosidase 4 [EC:3.2.1.37]	XYL4	

**Table 3:** DGEs enriched in the pathway of the MAPK signaling pathway—yeast.

Unigene ID	Expression							KEGG			MAPK–Yeast Pathway
	v9-1	v9-2	v26-1	v26-2	Log2FC	FDR	Status	KO	Description	Gene Name	
ctg12452_g1	2.42	4.83	0	0	5.237512	1.08 × 10^−2^	up-regulated	K02218	casein kinase 1 [EC:2.7.11.1]	CSNK1	pheromone to mating
ctg12155_g1	38.43	38.24	226.93	221.38	−2.55246	1.56 × 10^−247^	down-regulated	K04627	pheromone a factor receptor	STE3	
ctg11329_g1	16.3	14.63	0.91	1.3	3.79796	1.08 × 10^−51^	up-regulated	K19838	GTPase-activating protein SST2	SST2	
ctg7267_g1	10	8.02	4.22	2.29	1.629585	1.66 × 10^−15^	up-regulated	K19860	guanine nucleotide-binding protein alpha-1 subunit	GPA1	
ctg9995_g1	13.75	15.65	4.19	4.95	2.564613	2.47 × 10^−97^	up-regulated				
ctg7524_g1	15.49	16.67	2.44	2.49	2.650679	2.99 × 10^−13^	up-regulated				
ctg7524_g2	10.46	11.13	1.34	1.17	3.036185	2.17 × 10^−10^	up-regulated				
ctg12751_g1	3.75	2.25	0	0	5.071841	1.86 × 10^−2^	up-regulated	K07973	guanine nucleotide-binding protein subunit gamma	GNG	
ctg8861_g1	83.56	78.85	24.95	28.65	1.378415	3.00 × 10^−76^	up-regulated	K19839	Rho-type GTPase-activating protein 1/2	RGA1_2	
ctg7642_g1	6.45	6.09	34.3	36.89	−2.33243	3.27 × 10^−101^	down-regulated				
ctg4782_g1	2.79	2.23	7.26	5.49	−1.3039	4.97 × 10^−3^	down-regulated	K04393	cell division control protein 42	CDC42	
ctg3200_g1	7.21	5.98	1.42	1.82	2.014258	1.20 × 10^−20^	up-regulated	K11237	bud emergence protein 1	BEM1	
ctg2668_g1	2.6	4.31	0	0	5.071842	1.86 × 10^−2^	up-regulated	K04409	p21-activated kinase 1 [EC:2.7.11.1]	PAK1	
ctg7848_g1	8	6.52	2.17	1.32	1.930004	2.49 × 10^−7^	up-regulated	K19833	serine/threonine-protein kinase CLA4 [EC:2.7.11.1]	CLA4	
ctg8443_g1	40.03	38.71	16.05	20.1	1.123039	2.04 × 10^−16^	up-regulated	K19842	RHO1 GDP-GTP exchange protein 1/2	ROM1_2	cell wall stress to remodeling
ctg6770_g1	31.02	33.24	0.88	1.03	5.014033	1.57 × 10^−33^	up-regulated				
ctg9497_g3	4.38	3.34	1.49	1.15	1.517745	1.26 × 10^−2^	up-regulated				
ctg293_g1	1.57	3.72	0.95	0.27	2.007466	1.79 × 10^−2^	up-regulated	K19844	GTPase-activating protein BEM2	BEM2	
ctg4687_g1	3.52	2.64	6.67	7.29	−1.19529	6.18 × 10^−24^	down-regulated	K00888	phosphatidylinositol 4-kinase A [EC:2.7.1.67]	PI4KA	
ctg5599_g1	4.81	4.57	20.91	18.6	−2.09223	6.61 × 10^−47^	down-regulated	K11229	mitogen-activated protein kinase kinase kinase [EC:2.7.11.25]	BCK1	
ctg5657_g4	0.92	0	3.77	2.93	−2.70834	6.18 × 10^−2^	down-regulated	K19806	tyrosine-protein phosphatase 2/3 [EC:3.1.3.48]	PTP2_3	
ctg380_g1	0.62	0.62	3.93	4.62	−2.61052	1.05 × 10^−2^	down-regulated	K06276	3-phosphoinositide dependent protein kinase-1 [EC:2.7.11.1]	Pkh1	
ctg8570_g3	3.61	3.22	1.16	0.84	1.729908	5.36 × 10^−6^	up-regulated				
ctg1020_g1	11.05	11.08	4.74	5.05	1.171861	2.94 × 10^−6^	up-regulated	K11246	SHO1 osmosensor	SHO1	high osmolarity to osmolyte synthesis
ctg9076_g2	2.22	1.33	5.92	5.44	−1.68952	7.79 × 10^−11^	down-regulated	K11233	osomolarity two-component system, response regulator SSK1	SSK1	
ctg10627_g1	79.42	69.73	33.43	35.06	1.139585	3.06 × 10^−71^	up-regulated				
ctg5925_g2	3.98	4.16	0.56	0.25	3.106293	7.58 × 10^−7^	up-regulated	K06666	general transcriptional corepressor TUP1	TUP1	
ctg8863_g1	27.22	28.43	5.57	9.01	2.427738	4.45 × 10^−73^	up-regulated	K03114	mitosis inhibitor protein kinase SWE1 [EC:2.7.11.1]	SWE1	
ctg8944_g1	60.33	48.42	125.54	116.41	−1.17943	7.93 × 10^−52^	down-regulated	K03781	catalase [EC:1.11.1.6]	Ctt1	
ctg5701_g1	43.23	42.24	423.49	411.39	−3.30667	0	down-regulated	K09448	transcriptional enhancer factor	TEAD	starvation to filamentation
ctg6107_g1	22.31	23.76	4.29	2.92	2.639995	1.36 × 10^−46^	up-regulated				

**Table 4:** DGEs enriched in the pathway of the ABC transporters.

Unigene ID	Expression							KEGG		
	v9-1	v9-2	v26-1	v26-2	Log 2-FoldChange	FDR	Status	KO	Description	Gene Name
ctg2182_g1	547.5	525.1	237.3	243	1.139132	4.86 × 10^−202^	up-regulated	K05658	ATP-binding cassette, subfamily B (MDR/TAP), member 1 (EC:3.6.3.44) xenobiotic-exporting ATPase	ABCB1
ctg11186_g1	266.3	259.7	57.76	60.5	2.134296	0	up-regulated			
ctg11186_g2	274.3	252.4	50.93	61.7	2.235691	2.83 × 10^−179^	up-regulated			
ctg10041_g1	53.11	54.03	18.96	19.3	1.450944	1.56 × 10^−152^	up-regulated			
ctg11158_g1	29.92	26.85	11.12	11.8	1.290507	5.02 × 10^−79^	up-regulated			
ctg1141_g1	2.79	5.54	0	0	5.237513	1.08 × 10^−2^	up-regulated			
ctg3266_g2	1.73	0.58	3.16	8.03	−2.13418	1.2 × 10^−2^	down-regulated			
ctg4538_g1	0.81	0.41	4.22	4.44	−2.76898	2.43 × 10^−7^	down-regulated			
ctg8579_g1	111.7	102.4	289.9	290	−1.45923	3.27 × 10^−141^	down-regulated			
ctg11112_g1	30.84	25.83	71.78	75.5	−1.4194	2.98 × 10^−157^	down-regulated			
ctg7752_g1	26.95	26.32	53.58	54.9	−1.05833	5.22 × 10^−67^	down-regulated			

## Supplementary Materials

Supplementary materials can be found at https://www.mdpi.com/2073-4425/11/2/161/s1.
